# Antibiotic prescriptions associated with a diagnosis of acute nasopharyngitis by general GPs in France: a retrospective study

**DOI:** 10.3399/BJGPO.2024.0006

**Published:** 2024-10-16

**Authors:** Tran Tue Duong, Matta Matta, Beranger Lekens, Sylvain Diamantis

**Affiliations:** 1 Service de Maladies Infectieuses et Tropicales, Centre Hospitalier de Melun, Melun, France; 2 Cegedim SA, Boulogne-Billancourt, France; 3 Dynamic Research Unit, Université Paris Est Créteil, Créteil, France

**Keywords:** general practice, drug prescriptions, nasopharyngitis, amoxicillin, antibiotics

## Abstract

**Background:**

Nasopharyngitis is a common viral infection that has led to an overuse of prescription drugs, in particular antibiotics, which are not indicated for this condition.

**Aim:**

The purpose of this study was to describe drug prescriptions for patients with a diagnosis of acute rhinopharyngitis in general practices in France.

**Design & setting:**

Retrospective study of 1 067 403 prescriptions for a diagnosis of nasopharyngitis issued by 2637 physicians to 754 476 patients living in metropolitan France.

**Method:**

The data were sourced from the prescription software, Cegedim, for the period 1 January 2018 to 31 December 2021 and analysed according to patients’ and physicians’ ages.

**Results:**

A total of 2 591 584 medications were prescribed by GPs, with a median of three medications per patient. A total of 171 540 courses of antibiotics were prescribed (16% prescription rates), with amoxicillin being the most frequently prescribed (102 089 prescriptions; 59.5% of antibiotic prescriptions). Amoxicillin prescription increased in extreme age groups (18.2% of visits in those aged 9 years and under, and 10.0% of visits in those aged over 80 years, while patients aged 20–29-years were prescribed amoxicillin in just 2.9% of visits), and more prescriptions are issued by older doctors (GPs older than 70 years prescribed antibiotics in 26.4% of visits versus 3.2% of visits by GPs aged under 29 years).

**Conclusion:**

Nasopharyngitis is frequently a cause of therapeutic over-prescriptions including antibiotics, with an antibiotic prescription rate of 16%. Additional research is required to enhance our understanding of factors linked to drug prescriptions.

## How this fits in

Nasopharyngitis is a prevalent self-limiting disease of viral origin. Antibiotics are often prescribed even though they are not indicated. We aim to identify the characteristics of the physicians prescribing antibiotics for these patients, in order to establish a dedicated programme of antimicrobial stewardship.

## Introduction

Antimicrobial resistance (AMR) is a global health problem.^1^ It is estimated that by 2050 it will be responsible for 10 million deaths per year.^1^ Antibiotic consumption is responsible for the increase in AMR both at an individual and community level.^2–4^

In France, 90% of all antimicrobials intended for human health are prescribed in ambulatory care, with GPs responsible for 70% of those prescriptions.^5,6^ It is estimated that the majority of antimicrobials prescribed in ambulatory care are for the treatment of upper respiratory tract viral infections, particularly nasopharyngitis (NP).^7,8^ These prescriptions are typically based on clinical assessments rather than microbiological diagnoses. Overprescribing antibiotics for community-acquired respiratory infections such as NP has become a significant challenge for antimicrobial stewardship,^9^ even in diseases where studies have clearly shown limited efficacy of such treatments.^10,11^

The common cold, or NP, is a common benign self-limiting disease. Most prescribed medications, such as antibiotics, have either limited efficacy or are ineffective.^12^ French guidelines recommend prescribing paracetamol (acetaminophen) and/or decongestant as symptomatic treatment for NP.^13^ Despite these guidelines, most GPs prescribe a combination of medications for a patient with NP.^13^

Few studies have evaluated the management of acute NP by GPs and their compliance with published guidelines. To better understand antimicrobial usage by GPs, and establish better antimicrobial stewardship programmes as well as advise policy makers, this study aimed to analyse trends in medication prescriptions, particularly antibiotics, and the evolution of these trends over a 4-year period.

## Method

### Study design and participants

This was a physician-based registry survey based on the French version of The Health Improvement Network (THIN) database, an ambulatory medical prescription software (GERS Patient Data). GERS Patient Data is a large national database that has been collecting data since 1994 from computerised prescriptions from more than 3000 GPs using Crossway software and the MLM (monLogicielMedical.com) full web solution. Medical records are collected at the physician level and are coded according to the 10th Revision of the International Classification of Diseases (ICD-10) codes.^14^ The GPs are located throughout mainland France and are responsible for the care of more than 3.9 million active patients. Participating GPs were selected to be representative of all French GPs and stratified by age, sex, and location. THIN includes data per patient on prescribed medicines and their reimbursement.

### Data retrieval

From 1 Jan 2018 to 21 Dec 2021, we collected data on acute NP cases (ICD code = J00), retrospectively and anonymously, both at the patient and GP levels. Every entry by a GP with an ICD code of J00 was collected in our database. It was not possibile to verify GPs diagnoses. Data on the age of the patients as well as data on any medication prescribed associated with such diagnosis were also collected. As the data were anonymised, we were not able to collect data such as the postcodes of GPs or the gender of patients or GPs. The age of the GPs was also collected as a proxy for their years of experience.

### Statistical analysis

We used the observed proportions comparison test for all tests, with a two-tailed *P* value <0.05 considered as statistically significant. Statistical analysis was done using EPIINFO (version 7.1).

### Ethical statement

The THIN database obtained approval from the French National Data Protection Authority for data collection in 2002. It comprises fully anonymised electronic medical records compliant with the European general data protection regulations. As the study was a retrospective analysis using only secondary anonymised patient data without reporting on personal information, French legislation did not require additional ethical approval and subsequently the requirement for obtaining informed consent was waived.

## Results

### Population and general information

The study included 2637 GPs who used the prescription software. Of these, 1032 were female (38.8%) and 1605 (61.2%) were male. The median age was 60 years (interquartile range [IQR] 44–62 years).

GPs diagnosed 1 067 403 cases of NP in 754 476 unique patients. Of these, 413 012 (54.7%) were female. The patients’ median age was 23 years (IQR 6–52 years). The most represented age group was 0–9-year-olds (198 866; 26.4%). Children aged 0–18 years represented 40% (423 231) of consultations for NP. The number of cases of NP observed decreased by a third in 2020, then increased in 2021 (2018 = 319 550; 2019 = 321 414 [+0.5%]; 2020 = 207 571 [-35%]; 2021 = 218 868 [+5%]).

### Prescriptions

GPs issued prescriptions for a total of 2 591 584 medications with a median of three medications prescribed per consultation. The most frequently prescribed therapeutic agents are detailed in Table 1.

**Table 1.** Types of drugs prescribed for patients with nasopharyngitis between January 2018 and December 2021, and their percentage of total drugs prescribed for these patients

Drug	Number of prescribed medications	% of the total prescribed medication volume for these patients	95% confidence interval
Paracetamol/acetaminophen	498 624	19.24	19.19 to 19.29
Nasal steroids	475 424	18.34	18.29 to 18.39
Cough medications	454 896	17.55	17.50 to 17.60
Nasal vasoconstrictors	116 737	4.50	4.47 to 4.53
Antihistamines	114 029	4.39	4.37 to 4.41
Non-steroidal anti-inflammatory drugs	113 146	4.36	4.33 to 4.39
Penicillins	112 011	4.32	4.30 to 4.34
Systemic steroids	68 314	2.63	2.61 to 2.65
Macrolides	40 762	1.57	1.55 to 1.59
Cephalosporins	18 769	0.72	0.71 to 0.73
Other medications	578 872	22.33	22.28 to 22.38
Total	2 591 584	100	–

Paracetamol (acetaminophen) was the most widely prescribed, being present on 498 624 (46.7%, 95% confidence interval [95% CI] = 46.61 to 46.79) prescriptions, followed by tixocortol with 362 130 (33.9%, 95% CI = 33.81 to 33.99) prescriptions and cough syrup with 359 717 (33.7%, 95% CI = 33.61 to 33.79) prescriptions. Antibiotics were prescribed in 171 540 (16.1%, 95% CI = 15.93 to 16.07) visits.

In slightly over a quarter of the consultations (278 560, 26.1%, 95% CI = 25.91 to 26.08), GPs prescribed at least four medications, and in 267 437 (25.1%, 95% CI = 24.91 to 25.08) consultations, three medications were prescribed. In only 42 693 cases (3.9%, 95% CI = 3.96 to 4.03) did GPs not prescribe any medication.

The total number of prescriptions drastically decreased in 2020 and then slightly rebounded in 2021 (2018 = 810 434; 2019 = 773 596 [-4.5%]; 2020 = 482 120 [-38%]; 2021 = 525 434 [+9%)]).

We compared the mean number of prescriptions per patient age groups and GP age groups. We found that patients in the 20–29 year age group were prescribed more medications than any other group. Furthermore, the older the patient was, the fewer medications they were prescribed (Table 2).

**Table 2.** Percentage of prescriptions for amoxicillin and mean of medications prescribed by patient’s age

Patient’s age, years	Number of consultations	Number of prescriptions for amoxicillin (%) (95% CI)	Number of prescriptions for any medication (mean) (95% CI)
0–9	291 239	53 005 (18.2) (18.13 to 18.27)	718 803 (2.4) (2.457 to 2.463)**
10–19	132 056	16 771 (12.7) (12.64 to 12.76)*	346 116 (2.6) (2.616 to 2.624)**
20 to 29	105 407	3159 (2.9) (2.87 to 2.93)*	284 915 (2.7) (2.695 to 2.705)
30–39	112 757	4141 (3.6) (3.65 to 3.64)*	293 288 (2.6) (2.595 to 2.605)**
40–49	104 212	5072 (4.8) (4.76 to 4.84)*	259 824 (2.4) (2.486 to 2.494)**
50–59	109 561	5599 (5.1) (5.06 to 5.14)*	255 588 (2.3) (2.326 to 2.334)**
60–69	100 358	5969 (5.95) (5.90 to 6.00)*	218 808 (2.1) (2.176 to 2.184)**
70–79	72,513	4335 (5.98) (5.93 to 6.03)*	142 569 (1.9) (1.957 to 1.963)**
80–89	3409	3522 (10.3) (10.24 to 10.36)*	62 794 (1.8) (1.836 to 1.844)**
Total	1 067 403	102 089 (9.5)	2 591 584 (2.4)

*P<0.001 (compared to 0–9 year-old group). **P<0.001 (compared to 20–29 year-old group). CI= confidence interval.

We also found that very young physicians prescribed more medications (of any class) than other GPs, while physicians aged 70 years or older prescribed the fewest medications (Table 3).

**Table 3.** Percentage of antibiotics and mean of medications prescribed by doctor’s age

Doctor’s age, years	Number of consultations	Number of prescriptions for antibiotics (%) (95% CI)	Number of prescriptions for any medication (mean) (95% CI)
20–29	2539	80 (3.2) (3.17 to 3.23)	6368 (2.5) (2.457 to 2.543)
30–39	111 367	9072 (8.1) (8.05 to 8.15)*	256 744 (2.3) (2.296 to 2.304)**
40–49	198 011	21 392 (10.8) (10.74 to 10.86)*	478 269 (2.4) (2.407 to 2.413)**
50–59	340 290	56 353 (16.6) (16.53 to 16.67)*	835 258 (2.4) (2.453 to 2.457)**
60–69	383 392	76 247 (19.9) (19.82 to 19.98)*	941 403 (2.4) (2.453 to 2.458)**
≥70	31 804	8396 (26.4) (26.32 to 26.48)*	71 913 (2.2) (2.252 to 2.268)**
Total	1 067 403	171 540 (16.0)	2 591 584 (2.4)

*P<0.001 (compared to 20–29 year-old group).**P<0.001 (compared to 20–29 year-old group). CI = confidence interval.

Analysing all prescriptions, we found that of the 2 591 584 medications prescribed, 2 044 345 (78.8%, 95% CI = 78.78 to 78.82) were either not recommended or were contraindicated according to the French national guidelines.^13^

### Antibiotics

**Figure 1.**
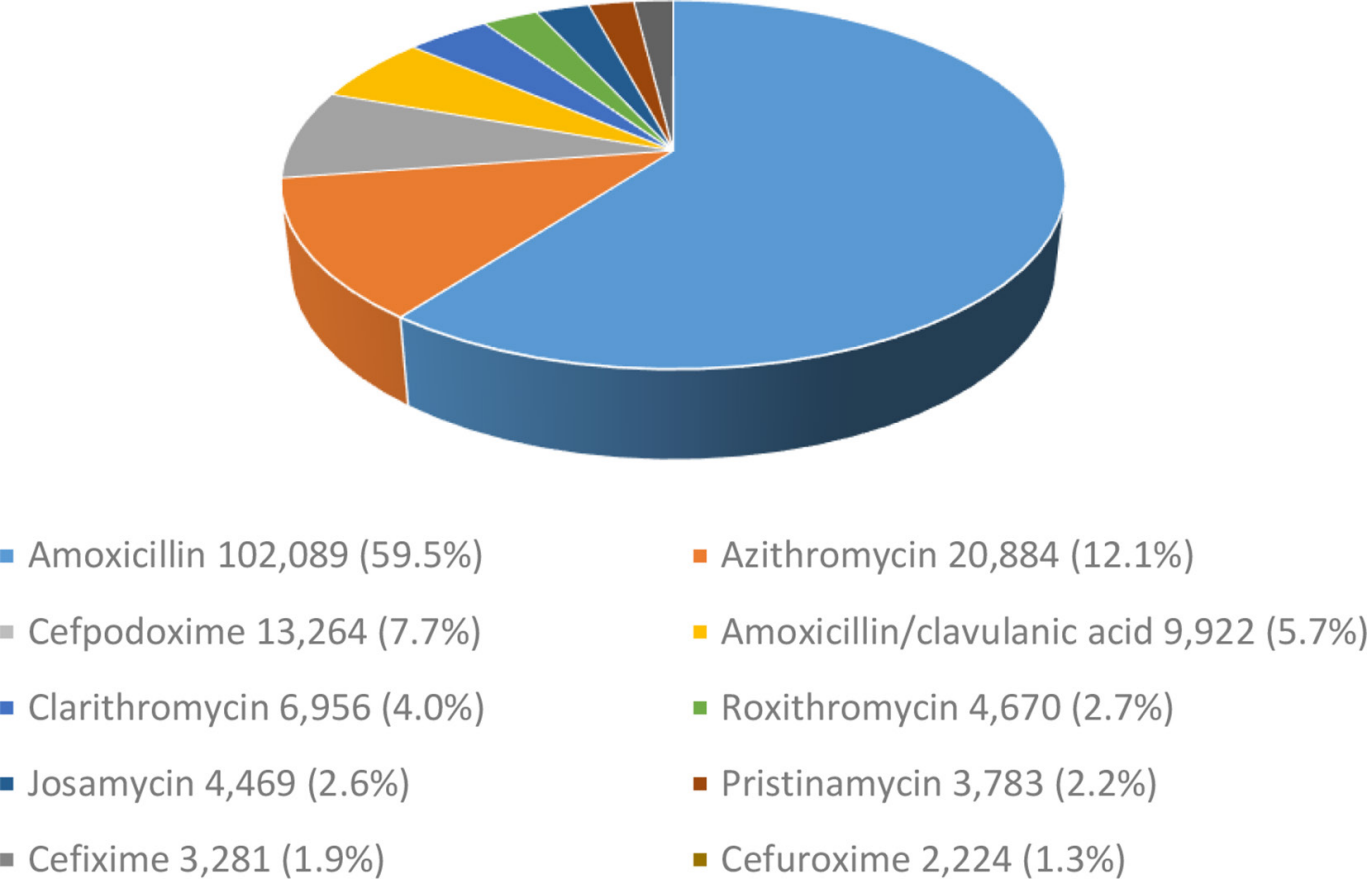
Antibiotic prescriptions for patients with nasopharyngitis (total number and percentage).

Antibiotics were prescribed in 16.1% (171 540) of cases over the 4 years covered by this study. Amoxicillin was the most prescribed antibiotic, with 102 089 prescriptions (59.5% of all antibiotics), and is the fourth most prescribed drug for patients with NP (Table 1). We also evaluated the use of antibiotics according to the AWaRe classification, which evaluates the impact of antibiotics on antimicrobial resistance.^15^ Antibiotics from the access group (narrow-spectrum antibiotics recommended as first and second choice for most common clinical infections) made up 65.2% of prescriptions, mostly amoxicillin (59.5% of all antibiotics or 102 089 prescriptions, 95% CI = 59.27 to 59.73) and amoxicillin and clavulanic acid (9922 prescriptions, 5.7%, 95% CI = 5.59 to 5.81). Antibiotics from the watch group (broader spectrum antibiotic classes) accounted for 34.8% (59 529, 95% CI = 34.58 to 35.02) of all antibiotics prescribed. From the watch group, azithromycin was most frequently prescribed (20 884 prescriptions, 12.1%, 95% CI = 11.95 to 12.25) followed by cefpodoxime (13 264 prescriptions, 7.7%, 95% CI = 7.57 to 7.83), clarithromycin (6956 or 4.0%, 95% CI = 3.90 to 4.10), roxithromycin (4670 or 2.7%, 95% CI = 2.62 to 2.78) and pristinamycin (3,783 or 2.2%, 95% CI = 2.13 to 2.27). No antibiotics from the reserve group (last resort antibiotics targeted for multidrug resistant infections) were prescribed (Figure 1).

**Figure 2.**
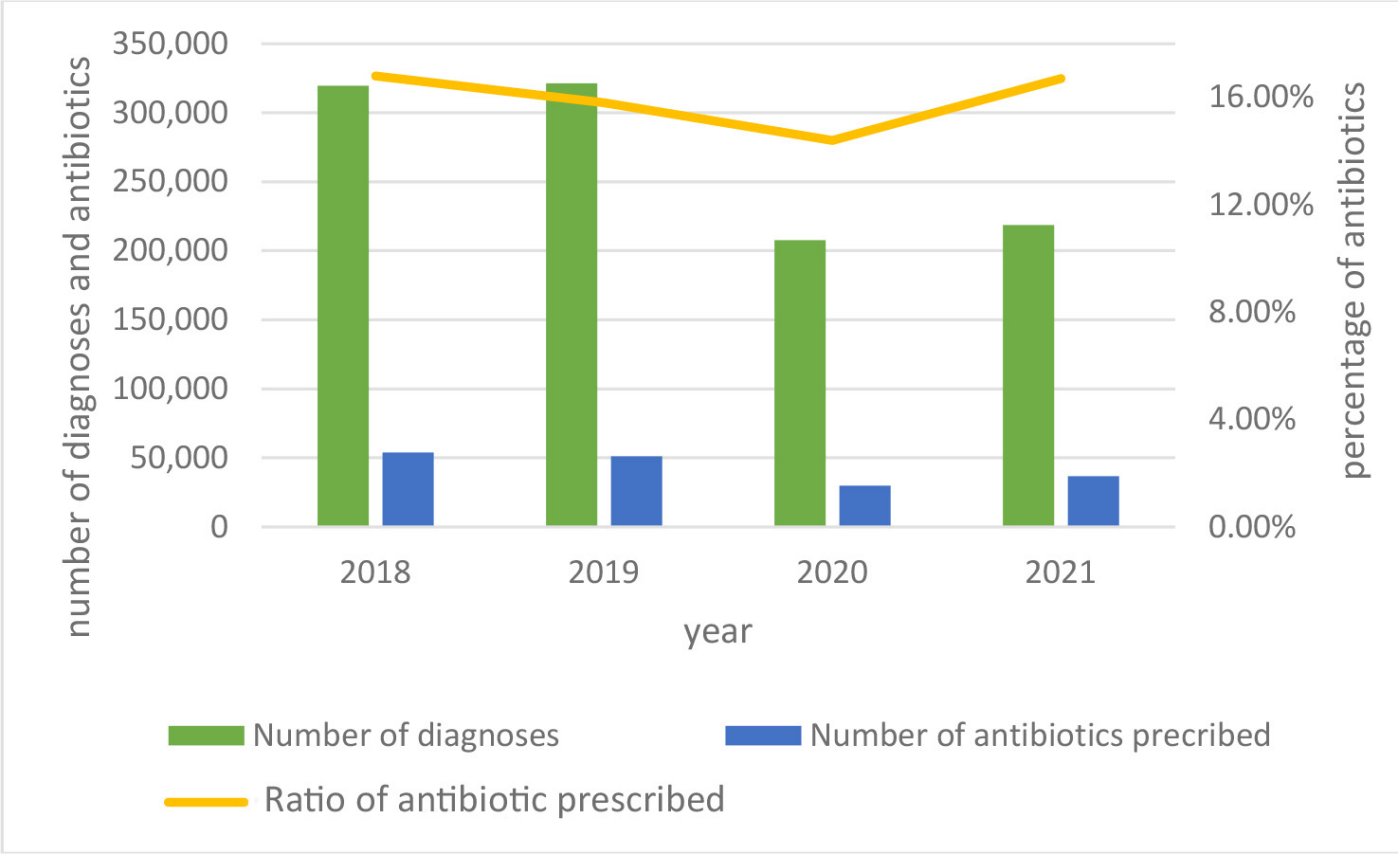
Antibiotic prescriptions 2018–2021.

Antibiotic prescriptions decreased each year from 2018 to 2020 before increasing again in 2021 (2018 = 53 898 prescriptions; 2019 = 51 070 prescriptions (-5.3%); 2020 = 29 955 prescriptions (-41.4%) because of the decrease of diagnosis of NP during the COVID-19 pandemic, then increased in 2021 = 36 617 prescriptions (+22%)) (Figure 2). The ratio of antibiotics prescribed to the number of visits decreased each year from 2018 to 2020 but returned to baseline in 2021 (2018 = 16.8%; 2019 = 15.8% (-6%); 2020 = 14.4% (-8.9%); 2021 = 16.7% (+16%) (Figure 2).

We could only retrieve amoxicillin prescription rates per patients’ age group; information could not be retrieved for other classes of drugs. Those at extremes of age were prescribed amoxicillin more frequently than other age groups, with children aged between 0 and 9 years old being prescribed the most (Table 2).

We found a significant association between the age of the prescribing physician and the prescription of antibiotics (*P*<0.001) with a correlation coefficient *r* = 0.96. The youngest physicians prescribed the fewest antibiotics but more non-antibiotic medication overall; and older GPs prescribed more antibiotics but fewer other medications. The older the physician, the higher the probability that they would prescribe antibiotics (Table 3).

## Discussion

### Summary

Our study showed that, between 2018 and 2021, antibiotics were prescribed by GPs in 16.1% of visits for NP. Furthermore, 80.7% of all prescribed medications were either not recommended or were contraindicated according to national guidelines.^13^ We also showed that amoxicillin prescription is more frequent in extreme age groups, and that younger GPs prescribed fewer antibiotics than older GPs. The increase in medication prescriptions is potentially associated with an increase in healthcare-associated costs as well as medication-associated adverse events.

### Comparison with existing literature

The 16.1% prescription rate for antibiotics in patients with NP in France in our study compares favourably with the prescription rates in the USA of 51% in an older study and 28% in a newer one, or the 49% prescription rate in a more recent study in Japan.^16–18^ The rate in France is considerably higher than the 4.6% prescription rate reported in the control group of an interventional study in Spain.^19^ Other studies evaluated antibiotic prescriptions in patients with upper respiratory tract infections and found a 38% prescription rate in neighbouring Belgium, while the rate in Sweden was 8%.^20,21^ The low rate in Sweden could be the result of adherence to national guidelines and dissemination of national recommendations on reducing the rate of antibiotic prescriptions in primary care.^21^

Very few studies have assessed the impact of symptomatic treatments for respiratory infections, either in terms of adverse effects or the cost burden of these prescriptions, and most of these studies showed that there is a high socioeconomic burden associated with acute NP,^21,22^ even without considering patient self-medication, as most drugs used to treat symptoms are available over the counter. It is therefore important to tackle issues associated with overconsumption of both antibiotics and treatments that target symptoms.

Several studies have highlighted different factors leading to overprescription of antibiotics in patients with respiratory tract infections, such as GPs overestimating symptoms, clinical uncertainty, patients’ expectations and physicians’ eagerness to meet those expectations, and patients’ and physicians’ ages.^6,23–26^ Others factors include workload, time pressure, and out-of-hours care (where a physician working outside of usual hours may not have time to properly assess the patient).^12,27^

Strategies proven to reduce antibiotic prescribing include educational interventions for primary care providers, and mass media educational campaigns aimed at healthcare professionals and the public.^12,28^ A study by Ferrat *et al* showed that the group which received a 2-day seminar prescribed fewer antibiotics than the control group, with a lasting influence over 4.5 years. However, the intervention group prescribed more treatments for symptoms than the control group.^28^ Substituting antibiotics with treatments for symptoms could help to reduce the development of antimicrobial resistance, but does little to curb healthcare costs as more than 50% of prescriptions included at least three drugs. Prescribing more treatments for symptoms instead of antibiotics might give the patient a feeling of being cared for and taken into consideration, thereby benefitting the GP as well in terms of their relationship with the patient. This is likely the result of a conceptual issue with medical prescriptions in France. The country’s liberal primary care system is focused on curative care and prescription-based consumption. The example of NP is a useful model for addressing the problem of unjustified over-prescription of drugs. A non-prescription order such as that promoted by the French Ministry of Health could help by giving patients a prescription paper that acknowledges their symptoms without actually prescribing any medication.^29^ It would be important to determine the efficacy of such a method before advocating for its widespread use.

In a previous publication in 2020, we showed that GPs prescribed antibiotics to around 7% of patients with COVID-19.^30^ This rate of antibiotic prescription is significantly less than in patients with NP, although COVID-19 was a poorly understood and potentially more severe disease. We think that the routine use of polymerase chain reaction reduced diagnostic uncertainty and potentially lead GPs to avoid prescribing antibiotics in patients with COVID-19. It could also be that the extensive media coverage of COVID-19 led to increased awareness among the general population about the viral origin of the disease and the absence of any role for antibiotics in the management of COVID-19, easing the pressure on GPs to prescribe antibiotics in these patients.

### Strength and limitations

The major strength of this study is the size of the database. We were able to access and analyse more than 1 million prescriptions associated with a NP diagnosis from GPs all over France. However, the study also has several limitations. First, we were not able to access the complete records. Therefore, there could have been a diagnostic classification bias, as clinical diagnosis is not always clear, especially for infants, and GPs may have prescribed antibiotics for an additional diagnosis alongside NP. Second, we were not able to identify patients’ chronic diseases and comorbidities and any association of these with an antibiotic prescription. Third, drug associations could not be analysed. Finally, we have analysed prescription as a proxy for consumption. Although it is safe to assume that most patients have taken their medications as prescribed, the possible use of over-the-counter medications cannot be tracked in our study.

### Implications for research and practice

These findings raise the question about using diagnostic tests for presumed viral infection, while also enhancing clinical education, disseminating guidelines, and promoting learning, akin to the introduction of e-learning and communication materials during the COVID-19 pandemic. This approach serves to minimise diagnostic uncertainty and the treatment ambiguity that may ensue.

Additional studies will be required to enhance our understanding of the factors linked with NP prescriptions, in order to further reduce antibiotic prescriptions and improve antimicrobial stewardship.

It is also necessary to conduct further research on the factors related to the prescription of medications to treat symptoms, with the aim of improving general patient health and reducing overall costs.

Despite published guidelines, this study demonstrates that most GPs are overprescribing in the treatment of NP. Antibiotics, in particular, account for one in five overprescriptions. Further research is needed to identify the parameters associated with this overuse of drugs and to review training strategies. Reforming the health care system as well as educating GPs is essential to reduce medication overuse. Additionally, educating patients about medication use can help to move away from a culture that is overly reliant on drug prescription, in particular antibiotics.

## Data

The dataset relied on in this article is available from the corresponding author on reasonable request.

## References

[1] Review on Antibiotic Resistance (2016). Tackling drug-resistant infections globally: final report and recommendations: review on antimicrobial resistance.

[2] Goossens H, Ferech M, Vander Stichele R (2005). Outpatient antibiotic use in Europe and association with resistance: a cross-national database study. Lancet.

[3] Costelloe C, Metcalfe C, Lovering A (2010). Effect of antibiotic prescribing in primary care on antimicrobial resistance in individual patients: systematic review and meta-analysis. BMJ.

[4] European Centre for Disease Prevention and Control (2022). Antimicrobial resistance in the EU/EEA - Annual epidemiological report for 2021..

[5] Santé Publique France (2022). [Antibiotic consumption and antimicrobial ressitance in France in 2021: where are we?] Consommation d’antibiotiques et prévention de l’antibiorésistance en France en 2021: où en sommes-nous? (in French).

[6] Bara W, Brun-Buisson C, Coignard B, Watier L (2020). Outpatient antibiotic prescriptions in France: patients and providers characteristics and impact of the COVID-19 pandemic. Antibiotics.

[7] Lehur A, Arias P, Kopp A, de Pontfarcy A (2020). [Analysis of antibiotic prescription by sing a prescribing software, in France, by general practitionners] *Analyse des prescriptions d’antibiotiques des médecins généralistes, en France, à partir d’un logiciel de prescription* (in French). Médecine et Maladies Infectieuses.

[8] Agence Nationale de la Sécurité du Médicament (2017). [Evolution of antibiotic consumption in france between 2000 and 2015] L’évolution des consommations d’antibiotiques en France entre 2000 et 2015 (in French).

[9] Bertino JS (2002). Cost burden of viral respiratory infections: issues for formulary decision makers. Am J Med.

[10] Lemiengre MB, van Driel ML, Merenstein D (2018). Antibiotics for acute rhinosinusitis in adults. Cochrane Database Syst Rev.

[11] Spinks A, Glasziou PP, Del Mar CB (2013). Antibiotics for sore throat. Cochrane Database Syst Rev.

[12] DeGeorge KC, Ring DJ, Dalrymple SN (2019). Treatment of the common cold. Am Fam Physician.

[13] Cohen R (2011). [Systemic antibiotic chemotherapy in general practice for upper respiratoruy tract infection] Antibiothérapie par voie générale en pratique courante dans les infections respiratoires hautes (in French).

[14] World Health Organisation (2019). International Statistical Classification of Diseases and Related Health Problems (ICD).

[15] World Health Organisation (2021). AWaRe classification.

[16] Gonzales R, Steiner JF, Sande MA (1997). Antibiotic prescribing for adults with colds, upper respiratory tract infections, and bronchitis by ambulatory care physicians. JAMA.

[17] Araki Y, Momo K, Yasu T (2021). Prescription pattern analysis for antibiotics in working-age workers diagnosed with common cold. Sci Rep.

[18] Walsh TL, Taffe K, Sacca N (2020). Risk factors for unnecessary antibiotic prescribing for acute respiratory tract infections in primary care. Mayo Clin Proc Innov Qual Outcomes.

[19] Llor C, Hernández S, Cots JM (2013). [Doctors with Rapid tests significantly decrease antibiotic prescribing for the common cold] *Los Médicos que disponen de pruebas Rápidas disminuyen significativamente la prescripción de antibióticos en el resfriado común* (in Spanish). Rev Esp Quimioter.

[20] Tyrstrup M, Beckman A, Mölstad S (2016). Reduction in antibiotic prescribing for respiratory tract infections in Swedish primary care- a retrospective study of electronic patient records. BMC Infect Dis.

[21] Tyrstrup M, van der Velden A, Engstrom S (2017). Antibiotic prescribing in relation to diagnoses and consultation rates in Belgium, the Netherlands and Sweden: use of European quality indicators. Scand J Prim Health Care.

[22] Jaume F, Valls-Mateus M, Mullol J (2020). Common cold and acute rhinosinusitis: up-to-date management in 2020. Curr Allergy Asthma Rep.

[23] O’Connor R, O’Doherty J, O’Regan A, Dunne C (2018). Antibiotic use for acute respiratory tract infections (ARTI) in primary care; what factors affect prescribing and why is it important? A narrative review. Ir J Med Sci.

[24] Teixeira Rodrigues A, Ferreira M, Roque F (2016). Physicians’ attitudes and knowledge concerning antibiotic prescription and resistance: questionnaire development and reliability. BMC Infect Dis.

[25] Akkerman AE, Kuyvenhoven MM, van der Wouden JC, Verheij TJM (2005). Determinants of antibiotic overprescribing in respiratory tract infections in general practice. J Antimicrob Chemother.

[26] Gjelstad S, Dalen I, Lindbaek M (2009). GPs’ antibiotic prescription patterns for respiratory tract infections--still room for improvement. Scand J Prim Health Care.

[27] Huibers L, Vestergaard CH, Keizer E (2022). Variation of GP antibiotic prescribing tendency for contacts with out-of-hours primary care in Denmark - a cross-sectional register-based study. Scand J Prim Health Care.

[28] Ferrat E, Le Breton J, Guéry E (2016). Effects 4.5 years after an interactive GP educational seminar on antibiotic therapy for respiratory tract infections: a randomized controlled trial. Fam Pract.

[29] l’Assurance Maladie (2023). [Viral infection, how to be treated?] Infection virale: comment vous soigner? (in French).

[30] Matta M, Gantzer L, Chakvetadze C (2024). Antibiotic prescription in ambulatory care for COVID-19 patients: a cohort analysis in four European countries. Eur J Clin Microbiol Infect Dis.

